# Wallerian degeneration of the corticospinal tract following multimodal high-grade glioma treatment: a case series and recommendations for radiotherapy planning

**DOI:** 10.3389/fonc.2025.1605190

**Published:** 2025-11-14

**Authors:** Monica D'Alma Costa Santos, Natasha Maranhão Vieira Rodrigues, Franceliny Gibram, Julia Brunelli, Ana Carolina Pinheiro Campos, Anselmo Mancini, Olavo Feher, Gustavo Nader Marta, Samir Abdallah Hanna, Caroline Chung, Fabio Ynoe Moraes, Marcos Vinicius Calfat Maldaun

**Affiliations:** 1 Department of Radiation Oncology, Hospital Sírio-Libanês, São Paulo, Brazil; 2 Medical School, Federal University of Amazonas, Manaus, Brazil; 3 Medical School, University of Sapucaí Valley, Pouso Alegre, Brazil; 4 Department of Radiology, Hospital Sírio-Libanês, São Paulo, Brazil; 5 Laboratory of Neuroscience, Hospital Sírio-Libanês, São Paulo, Brazil; 6 Department of Oncology, Hospital Sírio-Libanês, São Paulo, Brazil; 7 Department of Radiation Oncology, University of Texas MD Anderson Cancer Center, Houston, TX, United States; 8 Department of Radiation Oncology, Queen's University, Kingston, ON, Canada; 9 Department of Neurosurgery, Hospital Sírio-Libanês, São Paulo, Brazil

**Keywords:** high-grade glioma, corticospinal tract, Wallerian degeneration, radiotherapy, motor dysfunction

## Abstract

**Introduction:**

Wallerian Degeneration of the Corticospinal Tract (WDCT) is a scarcely reported complication of multimodal treatment for high-grade gliomas, which, despite its potential clinical impact, may lead to severe motor dysfunction and impair quality of life.

**Methodology:**

This retrospective case series describes 13 adult patients with high-grade gliomas treated between 2018 and 2023 who developed imaging findings consistent with WDCT after receiving multimodal treatment. Clinical and radiological data were collected from medical records; a standardized imaging follow-up or functional scoring system was not applied. Tractography was retrospectively available for two cases, enabling its incorporation into radiotherapy planning system for CST precise delineation and corresponding dose estimation.

**Results:**

WDCT was identified in 13 out of 192 high-grade glioma patients (6.8%). Diagnosis was based on T2/FLAIR hyperintensity along the ipsilateral CST with compatible clinical symptoms. Three cases developed WDCT after reirradiation, and seven (53.8%) had received bevacizumab prior to diagnosis: two during disease progression, two for the treatment of radionecrosis, and three as prophylaxis. Clinical symptomatology was detailed to 11 patients, 72.7% presented with hemiparesis, and 36.4% had seizures. In two cases, retrospective dose-volume analysis revealed CST mean doses ranging from 25.81 Gy to 42.83 Gy.

**Conclusions:**

This retrospective series highlights WDCT as a potentially underrecognized complication in glioma patients undergoing multimodal treatment. While etiology is likely multifactorial, radiotherapy may play a contributing role. CST delineation, when tractography is available, may support individualized treatment planning, enable better assessment of dose exposure, and help identify patients at higher risk of motor decline. Further prospective studies are warranted to define dose thresholds and assess functional outcomes.

## Introduction

The standard treatment for high-grade gliomas is multimodal, involving neurosurgery, radiotherapy, and chemotherapy (1). Initial management typically consists of maximal safe resection followed by radiotherapy with concomitant and adjuvant temozolomide, delivering a dose of 60 Gy (1), or 40 Gy for elderly patients (2). This comprehensive approach improved survival outcomes and made reirradiation a feasible option for selected cases (3). However, the infiltrative nature of gliomas presents significant therapeutic challenges, particularly in preserving critical brain regions, such as the motor cortex and associated white matter tracts, while ensuring adequate tumor coverage without compromising motor function and patient independence (4).

The corticospinal tract (CST) is a vital neural pathway originating from pyramidal neurons in the primary motor cortex, descending through various structures before decussating at the medullary pyramid to form the lateral CST (5, 6). Wallerian degeneration of the CST (WDCT), resulting from axonal injury, is initiated by excitatory neurotransmitters and proinflammatory cytokines, leading to microtubule disassembly, axonal swelling, and fragmentation. This process results in progressive motor impairment and can significantly affect quality of life (7).

Although WDCT has been described after neurosurgical intervention (8, 9), its occurrence following radiotherapy or as part of multimodal treatment for glioma remains underexplored. The present study reports a retrospective series of patients with high-grade gliomas who developed WDCT after multimodal therapy. It discusses potential mechanisms, describes associated clinical and radiological features, and explores the role of CST delineation in radiotherapy planning to mitigate functional compromise.

## Methods

This retrospective descriptive case series emerged from a review of all adult glioma cases treated at a Brazilian Radiation Therapy Department between January 2018 and December 2023. Out of 192 patients, 13 (6.8%) developed imaging and clinical findings consistent with WDCT. All patients had high-grade gliomas and underwent multimodal treatment. The study was approved by the Institutional Review Board (ID 3406).

The small sample size reflects the limited reporting of WDCT in glioma patients and underscores the exploratory nature of this analysis. Due to these limitations, survival outcomes such as progression-free survival (PFS) and overall survival (OS) were not evaluated. Instead, the analysis focused on demographic, clinical, systemic, and radiotherapy treatment data.

This report also proposes recommendations for CST contouring and highlights the importance of evaluating the dose delivered to this critical motor pathway, not with the intent of limiting treatment coverage or attributing motor deficits solely to radiotherapy, but to better understand its contribution within a multifactorial context. Such an approach may enhance the understanding of CST exposure and its potential association with motor dysfunction, thereby supporting safer and more informed radiotherapy planning.

Patient selection was based on clinical evaluation and characteristic imaging findings consistent with WDCT, assessed through standardized neuroradiological review. Clinical data were obtained retrospectively from medical records and did not involve standardized functional or quality-of-life scales, as evaluations were performed by different physicians during routine clinical practice.

**Figure 1:**
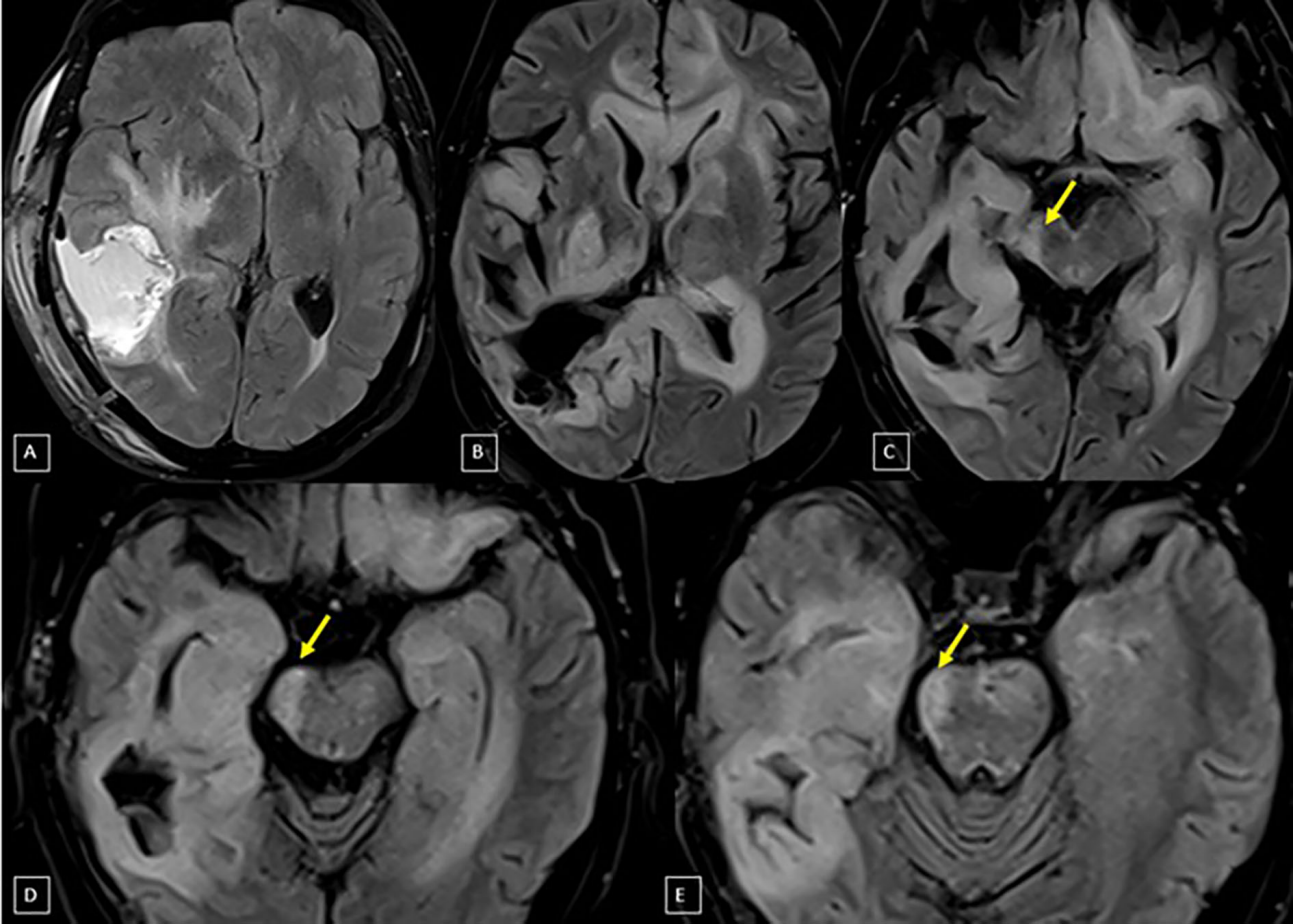
Axial plane MRI studies with FLAIR sequence: The first image **(A)** was taken post-surgery and before radiotherapy, showing the right temporoparietal surgical cavity and an adjacent area of signal alteration representing edema/tumor infiltration. The following images (**B–E** – superior to inferior) are from the follow-up, five years later, demonstrating volumetric reduction (arrows) affecting the right cerebral peduncle and midbrain tegmentum, extending to the right medullary pyramid, with an atrophic appearance likely related to treatment – suggestive of WDCT.

Similarly, imaging follow-up was not conducted according to a uniform protocol, but reflected the standard oncologic care provided to each patient. Imaging evaluation included Magnetic Resonance Imaging (MRI) with diffusion-weighted imaging (DWI) for all patients, and tractography when available. In all cases, WDCT was identified as T2/FLAIR (Fluid-Attenuated Inversion Recovery) hyperintensity along the expected anatomical trajectory of the CST, in the absence of contrast enhancement, mass effect, or diffusion restriction. These findings were stable or exhibited gradual progression followed by stabilization (**Figure 1**), documented in at least three sequential MRI studies with quarterly intervals in most cases, except for one patient who underwent only two MRI scans showing a similar pattern.

Tractography data were available for only two patients, in whom it was retrospectively integrated into the radiotherapy planning system to estimate the dose delivered to the CST. Delineation of this motor pathway was performed using Fiber Tracking software (10) based on DTI, enabling precise tract visualization and subsequent dosimetric analysis in these two cases.

To support the proposal for CST delineation, a consensus meeting was held with a multidisciplinary team, including a neuroradiologist, radiation oncologists, a clinical oncologist, and a neurosurgeon. The purpose was to ensure a unified understanding of CST delineation across specialties, considering both anatomical complexity and radiotherapy planning needs.

## Results

Thirteen patients with high-grade gliomas were included: seven (53.8%) were female and six (46.2%) males, with a median age of 64 years (29–73 years). Tumor lateralization revealed that 58.3% had right-sided tumors, 33.3% left-sided, and 8.3% bilateral involvement. All patients received standard radiochemotherapy with temozolomide; 12 received 30 fractions of 2 Gy, while one received 15 fractions of 2.7 Gy. Three patients developed WDCT after subsequent reirradiation. Bevacizumab was administered to seven patients (53.8%) prior to WDCT diagnosis: two during disease progression, three for the treatment of radionecrosis, and three as prophylaxis against radionecrosis.

Symptom data were available for 11 of the 13 patients, among whom eight (72.7%) presented with hemiparesis (three with brachial predominance, one with crural predominance), one (9.1%) with ataxia, and four (36.4%) experienced seizures, including one patient with both hemiparesis and seizures. Among the patients with hemiparesis, three were unable to walk independently, and five exhibited some degree of functional dependence. The patient presenting with ataxia also exhibited functional impairment. The median time from completion of initial radiochemotherapy to WDCT diagnosis was 10 months (range 2–32 months).

Detailed dosimetric data were available for two patients who had tractography-based CST delineation (**Table 1**).

**Table 1:** Dosimetric parameters of the CST in two patients with tractography-based delineation.

Patient	Tumor location	Fractions (Gy)	CST side	Volume (cc)	Mean dose (Gy)	Max dose (Gy)	Dose to 0.5cc (Gy)	V40 (cc)	V60 (cc)
1	Right frontoparietal	30 X 2.0 Gy	Ipsilateral	10.6	42.83	62.81	62.2	7.25	7.12
1	Right frontoparietal	30 X 2.0 Gy	Contralateral	18.5	21.54	61.77	51.0		
2	Right frontal	15 X 2.7 Gy	Ipsilateral	31.1	25.81	42.76	42.37	9.41	
2	Right frontal	15 X 2.7 Gy	Contralateral	30.3	14.05	35.18	24.19		

The table presents mean dose, maximum dose, and volume-dose metrics (V40, V60, and dose to 0.5 cc) for both ipsilateral and contralateral CSTs. All values were retrospectively estimated from radiotherapy treatment plans. V40 and V60 indicate the absolute CST volume (in cubic centimeters) receiving at least 40 Gy and 60 Gy, respectively.

**Figure 2:**
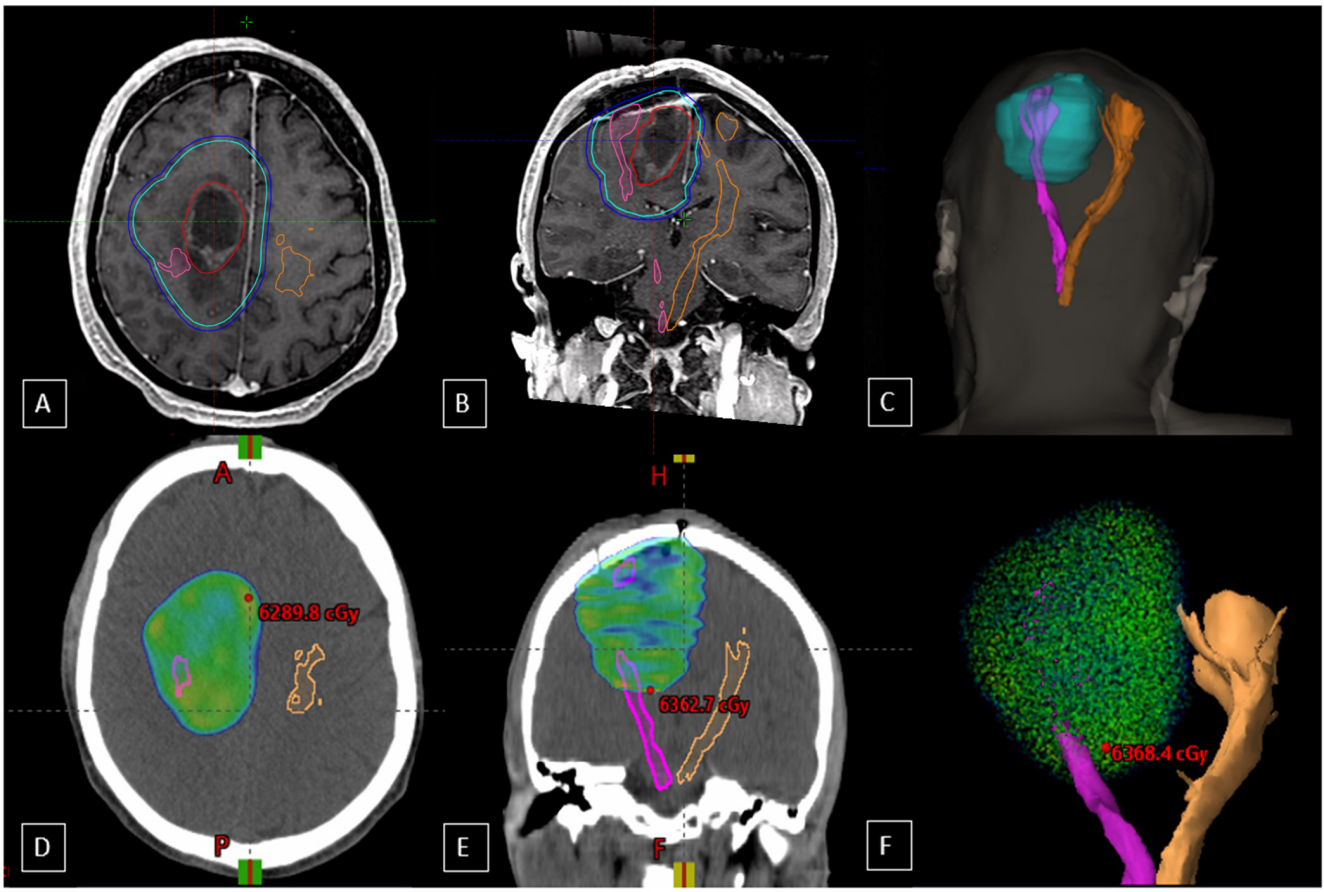
Axial **(A)** and coronal **(B)** T1-weighted images from the MRI fusion in the radiotherapy planning program, with the cyan blue line representing the CTV, the dark blue line indicating the PTV, and the red line outlining the GTV. The pink line outlines the right corticospinal tract (CST), and the orange line marks the left, contralateral CST. Image **(C)** is a 3D reconstruction illustrating the spatial relationship between target volumes and tracts. Axial **(D)** and coronal **(E)** views show the 60 Gy isodose distribution relative to the CTV and CST, highlighting dose exposure to surrounding structures. Image **(F)** provides a 3D reconstruction of the 60 Gy isodose distribution relative to the tracts, with the CTV encompassing the GTV plus a 1.5 cm margin and FLAIR hyperintensity.

Patient 1 had a right frontoparietal tumor and received 30 fractions of 2 Gy. The ipsilateral CST volume was 10.6 cc, receiving a mean dose of 42.83 Gy, a maximum dose of 62.81 Gy, and 0.5 cc receiving 62.2 Gy. The V60 was 7.12 cc, and the V40 was 7.25 cc. The contralateral CST volume was 18.5 cc, with a mean dose of 21.54 Gy, a maximum dose of 61.77 Gy, and 0.5 cc receiving 51 Gy (**Figure 2**).

Patient 2 had a right frontal tumor and received 15 fractions of 2.7 Gy. The ipsilateral CST volume was 31.1 cc, receiving a mean dose of 25.81 Gy, a maximum dose of 42.76 Gy, and 0.5 cc receiving 42.37 Gy, and the V40 was 9.41 cc. The contralateral CST volume was 30.3 cc, with a mean dose of 14.05 Gy, a maximum of 35.18 Gy, and 0.5 cc receiving 24.19 Gy.

## Discussion

Radiotherapy is a part of the multimodal treatment for gliomas but may result in collateral damage to surrounding healthy tissues, especially in eloquent brain areas like the CST (1, 2, 5). While the neurotoxic effects of radiation on white matter tracts such as the optic pathway are well-documented, the impact on motor pathways has been less explored (6). This case series highlights WDCT as a potential complication in patients with high-grade gliomas undergoing treatment and brings attention to a gap in the literature regarding CST vulnerability, particularly in the context of radiotherapy.

WDCT is a well-recognized sequela of axonal injury and is consistently associated with motor dysfunction, manifesting as weakness, loss of coordination, and spasticity (7). Although formal motor function scoring was not applied in this study, many patients exhibited functional dependence. The severity of motor impairment correlates with CST damage, underscoring the importance of assessing motor pathways before and after radiotherapy. In a recent prospective study, Connor et al. (11) evaluated fine motor skill decline following brain radiotherapy and found that higher doses to the corticospinal tract (CST) were significantly associated with functional deterioration. Specifically, both Dmax >53.5 Gy and Dmean >19.5 Gy to the CST correlated with worse performance on the pegboard test at 6 months post-treatment, with statistical significance across multiple analyses (p < 0.05). These findings reinforce the relevance of the CST as a dose-sensitive structure and highlight its potential impact on functional outcomes.

**Figure 3:**
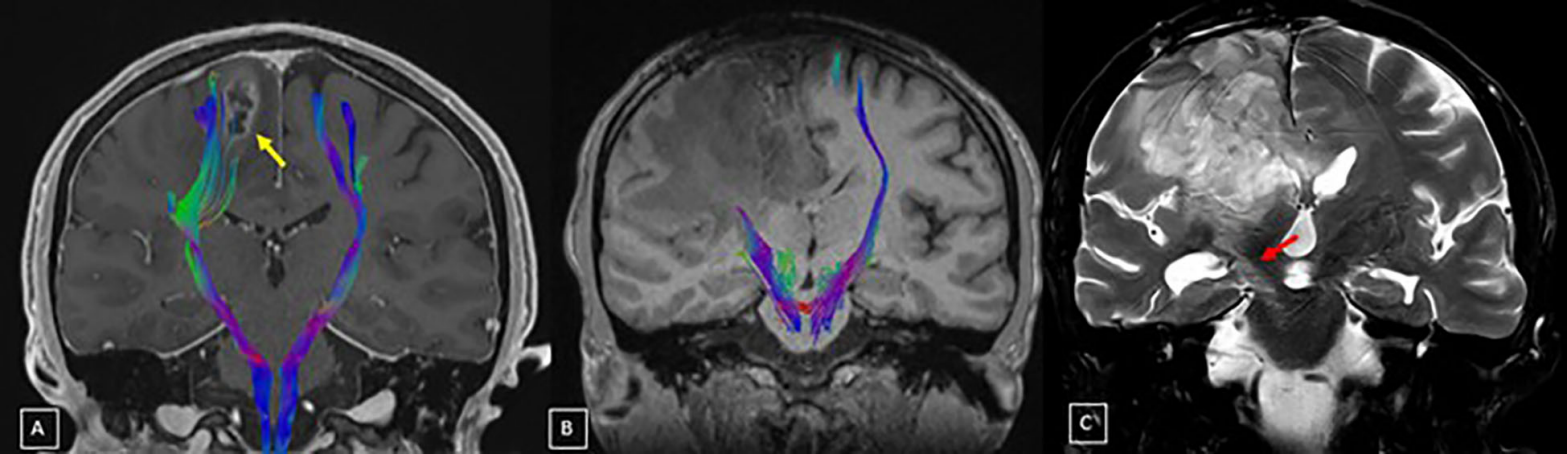
Pre-treatment baseline study **(A)**, tractography combined with post-contrast T1-weighted MRI in the coronal plane reveals a lesion in the right frontal lobe, with a necrotic center, irregular margins, and contrast enhancement (yellow arrow). In the tractography study **(A)**, there is asymmetry in the distribution of fractional anisotropy in the signal columns representing the right corticospinal tract, with posterior displacement. After treatment, a marked reduction in the fibers representing the topography of the right CST is noted **(B)**. A few months after treatment, coronal plane T2-weighted MRI **(C)** shows a signal alteration along the course of the right corticospinal tract, extending from the posterior limb of the internal capsule to the pons, characterized by T2/FLAIR hyperintensity, without significant retractile effect, diffusion restriction, or contrast enhancement, suggestive of WDCT (red arrow).

It is important to note that WDCT has a multifactorial etiology, with potential contributions from surgical resection, tumor tract involvement, and radiation-related axonal injury (**Figure 3**). Although a direct causal link cannot be established, studies suggest that radiation-induced axonal degeneration may result from oxidative stress and mitochondrial dysfunction. Ionizing radiation generates reactive oxygen species (ROS), such as superoxide and hydrogen peroxide, which damage cellular structures and disrupt homeostasis, especially in axons reliant on stable mitochondrial function. Oxidative stress may deplete NAD+ (Nicotinamide Adenine Dinucleotide), a coenzyme critical for cellular metabolism and activate SARM1 (Sterile Alpha and TIR Motif-Containing 1), leading to axonal disintegration and distal fragmentation. In addition, radiation can activate the MAPK (Mitogen-Activated Protein Kinase) pathway, impairing DNA repair and protein synthesis, further compromising neuronal and glial cell function, including myelin maintenance (12, 13). These mechanisms may contribute to the pathogenesis of WDCT in the context of multimodal treatment and reinforce the rationale for investigating a potential dose-response relationship involving CST exposure. Moreover, tumor infiltration of the CST itself may contribute to axonal degeneration and was not systematically evaluated in this study. Histopathological confirmation or precise spatial correlation between tumor progression and regions of degeneration was not available, representing a potential confounding factor.

In this series, tractography-based CST delineation was retrospectively available for only two patients, allowing estimation of the dose delivered to the tract, an approach still uncommon in routine radiotherapy planning due to the absence of specific dose constraints for this structure. These findings provide a preliminary basis for considering the CST as an organ at risk (OAR). When tractography is available, CST contouring should be encouraged (14), not necessarily to reduce target coverage or underdosing regions within the gross tumor volume (GTV) or clinical target volume (CTV), but to allow better evaluation of CST dose-volume exposure and to avoid hotspots when feasible.

This study presents several limitations. The small sample size and retrospective design limit generalizability and the lack of a comparator group (glioma patients who did not develop WDCT) limits the ability to differentiate which clinical, imaging, or treatment factors are truly associated with this complication, introducing further selection bias. Standardized functional scales (e.g., MRC, modified Rankin Scale) and quality-of-life instruments were not applied, limiting objective assessment of clinical impact; symptom evaluation was heterogeneous and dependent on non-standardized physician documentation. Additionally, three cases occurred after reirradiation, which may have influenced the development of WDCT. Although WDCT was identified in our cohort, its true frequency in glioma patients remains uncertain. The condition is rarely reported in the literature, which is limited to single cases or very small series and lacks denominator-based estimates.

Another important limitation is the lack of a standardized imaging follow-up protocol. Fractional anisotropy (FA) reduction on DTI, an early and sensitive marker of WDCT was not assessed, despite its potential to detect axonal injury prior to conventional MRI changes (15). In contrast, T2/FLAIR hyperintensity typically appears later, during the subacute or chronic phases (15) and may be attenuated in patients receiving antiangiogenic therapy. In our cohort, seven patients were treated with bevacizumab, which may have delayed or masked typical imaging findings. Finally, tractography was available for only two patients, limiting dose- volume retrospective correlation across the cohort. This reflects a broader challenge, as tractography adds cost and technical complexity, limiting its routine clinical use. Importantly, selection bias must also be acknowledged: cases were retrospectively identified based on the presence of clinical symptoms and imaging suspicion. While this strategy strengthens diagnostic confidence, it likely overrepresents symptomatic patients and under detects subclinical WDCT.

These limitations do not invalidate the relevance of the CST as a sensitive structure but underscore the need for prospective studies with standardized functional, radiological, and dosimetric assessments. According to the ESTRO-EANO guideline on radiotherapy for glioblastoma (16), dose constraints should serve as guidance for plan optimization; however, coverage of the Planned Target Volume (PTV) should not be compromised for non-critical structures. Although no specific constraints are currently established for CST coverage, delineation of the tract should be considered when tractography is available. At this stage, the goal is not necessarily to reduce the dose to the CST, but rather to estimate its exposure and evaluate possible clinical implications. CST delineation may also help identify patients at increased risk of motor dysfunction, enabling early interventions such as physical therapy, neuroprotective strategies, or timely rehabilitation.

### Recommendations for CST delineation in radiotherapy planning

- Neuroradiologist Involvement: Collaborate with a neuroradiologist to ensure precise CST delineation.
- Utilize DTI-Based Tractography: Use software capable of processing DTI data to generate a high-resolution CST visualization through tractography. This process analyzes water diffusion along axonal pathways, providing a detailed CST map.
- Integration with Radiotherapy Planning: Import delineated CST data into the radiotherapy planning system. Perform MRI and CT fusion (including tractography data) to achieve accurate spatial alignment of the CST within the treatment plan.
- Delineate as an Organ at Risk (OAR): Delineate the CST as an OAR. This designation will guide dose evaluation and help prevent excessive exposure. To enhance comparison and assessment, delineate both the ipsilateral and contralateral CSTs in the treatment planning.
- Avoid Hot Spots: When possible, avoid areas of high-dose accumulation (“hot spots”) within the CST. If these regions cannot be entirely excluded due to proximity to the CTV or PTV, minimize the exposure as much as possible.
- Dose Evaluation via Dose-Volume Histograms (DVHs): Assess dose distribution along the CST using DVHs. Minimize CST exposure by adjusting beam angles or intensity within the treatment plan.
- Post-Treatment Evaluation: Conduct a post-treatment MRI to assess any CST integrity changes, which can indicate treatment effects like WDCT.

## Conclusion

This case series draws attention to WDCT as a potentially underestimated complication in glioma patients undergoing multimodal treatment, with multifactorial contributions from surgery, tumor infiltration, and radiotherapy. Although definitive causality with radiotherapy cannot be confirmed, our findings highlight the CST as a functionally critical structure that may be susceptible to radiation-induced injury. The incorporation of CST delineation into radiotherapy planning, when tractography is available, may represent a practical step toward more personalized treatment approaches. This does not imply compromising target volume coverage but rather supports the assessment of dose exposure and identification of patients at higher risk for motor dysfunction. Further prospective studies are needed to establish validated dose constraints for the CST, integrate functional imaging tools, and evaluate long-term clinical outcomes.

## Data Availability

The datasets presented in this article are not readily available because The data supporting this study were collected under institutional ethical board approval and were not intended for public sharing. Access may be granted upon reasonable request to the corresponding author, subject to ethical and confidentiality restrictions. Requests to access the datasets should be directed to MS, monicadalmacosta@gmail.com.
